# COGNITION MEASURES AND FINDINGS IN THE NATIONAL SOCIAL LIFE, HEALTH, AND AGING PROJECT

**DOI:** 10.1093/geroni/igad104.0231

**Published:** 2023-12-21

**Authors:** Linda Waite, Dawn Carr

## Abstract

Good cognitive function is an important component of health at any age. Certain domains of cognition tend to decline with age and rates of change vary dramatically across individuals and across social groups. This Symposium examines a commonly-used clinical measure of cognition, the Montreal Cognitive Assessment, adapted for survey use (MoCA-SA) and administered in two rounds of the National Social Life, Health and Aging Project (2010 and 2015). It focuses especially identifying and evaluating differential functioning of the MoCA-SA across racial and ethnic groups, across modes of administration of the measure, within intimate dyads and as linked to sensory function. Iveniuk and colleagues examine race difference and find that 7 measures, out of the 18 used in NSHAP’s MoCA, formed a scale that was more robust to racial bias and suggest use of this modified measure to compare racial groups. Piedra and coauthors construct an abbreviated MoCA-SA (Spanish version) that compared favorably with the long form MoCA across the different grouping and showed predicative validity with consequential outcomes associated with cognitive decline. Pudelek and colleagues examine mode of assessment. web, phone, and PAPI or in-person interviews and describe an analytic strategy for obtaining a measure comparable across modes. Meiyi Li and Yiang Li find that women’s cognitive impairment adversely affects their partner’s social connectedness but husband’s impairment does not. Zhong et al find that odor identification was associated with some domains of cognition with differences in that association by age, gender, or race, but not by education.

